# The Important Roles Played in Substrate Binding of Aromatic Amino Acids in Exo-Inulinase From *Kluyveromyces cicerisporus* CBS 4857

**DOI:** 10.3389/fmolb.2020.569797

**Published:** 2020-09-25

**Authors:** Junyan Ma, Tang Li, Haidong Tan, Wujun Liu, Heng Yin

**Affiliations:** ^1^Natural Products and Glyco-Biotechnology Research Group, Liaoning Provincial Key Laboratory of Carbohydrates, Dalian Institute of Chemical Physics, Chinese Academy of Sciences, Dalian, China; ^2^Medical College, Dalian University, Dalian, China; ^3^Institute of Cancer Stem Cell, Dalian Medical University, Dalian, China

**Keywords:** exo-inulinase, inulin, glycoside hydrolase family 32, site-directed mutagenesis, substrate binding, molecular docking

## Abstract

Inulinase is a member of the glycoside hydrolase family 32 (GH32). It catalyzes the randomly hydrolyzation of 2,1-β-D-fructosidic linkages in inulin and plays a role in the production of high-fructose syrup. In this study, detailed roles of the conserved residues W79, F113, M117, R181, C239, and W334 of the exo-inulinase from *Kluyveromyces cicerisporus* CBS4857 (KcINU1) in substrate binding and stabilization were evaluated by *in silico* analysis and site-directed mutagenesis. These residues belong to the conserved WG, FSGSMV, RDP, ECP, and WQY regions of the GH32 and are located around the catalytic pocket of KcINU1. Zymogram assay showed relatively weaker band for F113W and similar band for M117A compared to the wild-type enzyme toward inulin and sucrose, whereas all other variants showed no observable stain on the native polyacrylamide gel electrophoresis. These results were further confirmed with the dinitrosalicylic acid colorimetric method. It showed that the residual activities of F113W toward inulin and sucrose were 33.8 ± 3.3% and 96.2 ± 5.5%, respectively, and that of M117A were 103.8 ± 1.3% and 166.5 ± 12%, respectively. Results from fluorescence spectra indicated that there is a significant conformational change that happened in F113W compared to the wild-type enzyme, while M117A exhibited limited impact although the quenching effect was increased.

## Introduction

Inulinase is a β-2,1-D-fructan fructanohydrolase, which targets the β-2,1 bond of inulin and hydrolyzes it into fructose (Gong et al., 2008). According to the mode of action on inulin, it can be classified into endo-inulinase (EC 3.2.1.7) and exo-inulinase (EC 3.2.1.80) (Arjomand et al., 2017). Exo-inulinase is a key enzyme used in *Jerusalem artichoke* bio-refinery. It hydrolyzes *J. artichoke* to fructose through one-step catalysis. The high purity fructose syrup produced can be used to produce ethanol fuel, butanol fuel, single-cell grease, biodiesel fuel, mannitol, and lactic acid. All products have great value in food, medicine, and bio-energy fields, etc. Therefore, the identification and development of inulinase has attracted great attention in the field of scientific research.

Inulinase belongs to the Glycoside Hydrolase (GH) family 32 in the Carbohydrate-active enzymes database^1^, and also includes invertase, levanase, eukaryotic fructan exohydrolase (FEH), and fructosyltransferase (FBE) (Pons et al., 2004; Lammens et al., 2009). The GH32 family has common 3D structural features, such as an N-terminal 5-fold beta-propeller forming the catalytic domain, and the C-terminal beta-sheet sandwich (Verhaest et al., 2005; Alberto et al., 2006; Bujacz et al., 2011). Each propeller at the N-terminus composed of four anti-parallel β-sheets, which form a classical “W” topology, radially surround the central axis, and enclose a negatively charged catalytically active pocket (Nagem et al., 2004).

Previous research regarding the structure of the GH32 family can be traced back to the 1990s. The early studies had mainly focused on determining the catalytic center of the enzymes by site-directed mutagenesis (Reddy and Maley, 1990; Sinnott, 1990). This was followed by the study of the roles played by amino acids in the conserved regions, as well as the amino acids around the active sites in the enzyme catalysis, steric stabilization, and substrate specificity (Reddy and Maley, 1996; Davies et al., 1997; Rye and Withers, 2000). By far, the structures of 16 types of GH32 family members have been determined^2^.

As a member of the GH32, there are six highly conserved sequences in exo-inulinase, namely WMNDPNG, RDP, ECP, SVEVF, FS, and Q (Liu et al., 2012). Except for SVEVF, these conserved sequences were determined to be located in the catalytic region of the N-terminal 5-fold beta-propeller. The three highly conserved acidic amino acids (D, D, and E) in the first three conserved regions (WMNDPNG, RDP, and ECP) are located at the bottom of the substrate binding pocket, forming the “catalytic triad,” responsible for the catalysis of the substrate (Lammens et al., 2009; Vandamme et al., 2013). The crystal structure of *Thermotoga maritima* invertase mutant E190D in complex with the trisaccharide raffinose [α-D-galactopyranosyl(1,6)-α-D-glucopyranosyl-β-D-fructofuranoside] (PDB code 1W2T) revealed that raffinose is bound between the blades of each β-propeller module, forming three substrate-binding sites −1, +1, and +2 with cleavage taking place between −1 and +1 (Alberto et al., 2006). The N, Q, and R in the conserved WMNEPNG, Q, and RDP regions are important for identifying and binding substrates, such as N322, Q339, and R459 in the *Arthrobacter* sp. S37 inulinase (Kim et al., 2008) and N40, Q57, and R188 in the *Aspergillus awamori* exo-inulinase (Nagem et al., 2004). The SVEVF sequence is located at the C-terminus of the β-sandwich region, where S is essential for maintaining the activity of the *Aspergillus niger* exo-inulinase InuE (Goosen et al., 2009). This sequence is located far away from the catalytic center, and is believed to play an important role in the binding of high molecular weight fructans (Goosen et al., 2009). However, the SVEVF motif was not observed in exo-inulinase produced by yeast (Liu et al., 2012). Furthermore, several amino acids are responsible for the specific recognition and binding of fructose residues (Alberto et al., 2006). There are three conserved aromatic residues (W41, F74, and W260) around the glucose binding sites, which form the wall of the substrate binding cleft (Alberto et al., 2006). Interestingly, none of them has established typical hydrophobic stacking interactions with the glucose unit, and has been thought to be a mechanism that allows adaptation to binding of other sugar units (Alberto et al., 2006). In addition, the galactose residue in the raffinose is located at the +2 site, adjacent to the surface part of the protein. This indicates that other sugar units of long-chain substrates may be suspended in the polar solvent instead of entering the active pocket (Liebl et al., 1998; Alberto et al., 2006; Lammens et al., 2009).

Natural source of exo-inulinase has difficulty meeting the requirements of industrial production. Previously, we have identified a novel exo-inulinase, named KcINU1, from *Kluyveromyces cicerisporus* and demonstrated the residues D53 and E238 (numbered D30 and E115, respectively, in previous paper which did not include the signal peptide sequence) involved in the catalytic activity of the enzyme (Ma et al., 2016). Further experiments showed that N-glycosylation significantly affected the enzymatic activity and thermostability of the enzyme (Ma et al., 2019). However, the enzyme-substrate interaction and catalytic mechanism is still unclear. In the present study, the binding region of the substrate was studied using bioinformatic analysis and site-directed mutagenesis. Eight variants were constructed using restriction-free (RF) cloning, including W79L, F113W/L, M117A, R181L/K, C239A, and W334L. After being expressed and purified in *Pichia pastoris* X-33 cells, the catalytic activity of these variants were evaluated by 3,5-dinitrosalicylic acid (DNS) assay and zymography study. Besides, fluorescence spectrometry study further elucidated the effects of the conserved amino acids on the conformation of the enzyme. The results obtained in this study will facilitate the future designing of tailored enzymes with better kinetics for meeting today’s practical needs.

## Materials and Methods

### Materials

Plasmid pPICZαA-rKcINU1 containing the *kcINU1* gene (GenBank accession number AF178979) without the N-terminal signal peptide coding sequence was preserved in this laboratory (Ma et al., 2016). Phusion Hot Start II DNA polymerase was purchased from Thermo Fisher (China). All primers were synthesized by BGI Company (China). All reagents were of analytical grade.

### *In silico* Analysis

The three-dimensional model of KcINU1 was obtained as described previously (Ma et al., 2019). The model was automatically built through the Swiss-Model server^3^ using the crystal structure of exo-inulinase INU1 (PDB code 6J0T) as the template which sharing 98.2% sequence identity with the KcINU1. Secondary structure alignment of KcINU1 and four other homology enzymes was performed using ESPript 3.0 (Robert and Gouet, 2014). The docking study was carried out using Tripos’ Sybyl X-2.1.1 software package. The ligand (1-kestose, raffinose, and sucrose) models for docking study were generated using BIOVIA Discovery Studio Visualizer software. The protein structure was energetically minimized using the Tripos force field (Clark et al., 1989) with default parameters. The Surflex-Dock (SFXC) docking mode was employed for molecular docking calculation and the binding pocket was defined through the automatic assignment with default parameters followed by manual inspection. Surf-Dock scores (total scores) were expressed in kcal/mol units to represent binding affinities. The ligand-protein interactions were visualized and analyzed using UCSF Chimera software (Pettersen et al., 2004). Plots showing the interaction between ligands and surrounding residues were prepared using the LIGPLOT+ version 1.4 program (Laskowski and Swindells, 2011).

### Site-Directed Mutagenesis, Protein Expression and Purification

Site-directed mutations were performed using RF cloning method the same as previously described (Ma et al., 2019). The pPICZαA-rKcINU1 recombinant plasmid was used as the template, and primers (Supplementary Table 1) used in RF-cloning were designed by online service tool^4^. PCR reactions were performed to generate mega-primers (200–600 bp) using Phusion Hot Start II DNA polymerase and the primer pairs listed in Supplementary Table 1 with the pPICZαA-rKcINU1 as the template. Then, second PCR reactions were carried out using the obtained mega-primers as the primer and the pPICZαA-rKcINU1 as the template. The final PCR products were digested with *Dpn*I (New England Biolabs) before transformed into *Escherichia coli* Top 10 competent cells. The obtained recombinant plasmids were verified by sequencing service provided by BGI (Beijing) with primers listed in Supplementary Table 1 before being linearized and transformed into *P. pastoris* by electroporation. The protein expression and purification was conducted as described previously (Ma et al., 2016). In brief, the selected recombinant *P. pastoris* X-33 cells were cultured in Buffered Glycerol-complex (BMGY) medium and the expression was induced by the addition of 0.5% (v/v) methanol in Buffered Methanol-complex (BMMY) medium. After 96 h induction with the additional supplement of 0.5% (v/v) methanol every 24 h, the supernatant was collected by centrifuge at 3000 rpm for 10 min. The target proteins were purified using Ni-NTA affinity column and their purities were evaluated by sodium dodecyl sulfate polyacrylamide gel electrophoresis (SDS-PAGE).

### Activity Analysis and Kinetic Parameters Determination

The procedure of zymogram analysis was conducted as previously described (Ma et al., 2016). In this study, sucrose and inulin were used as the substrates. After native PAGE separation at 4°C, the gel was completely immersed in a 2% (w/v) sucrose solution and incubated at 55°C for 1 h. Then, the gel was washed thoroughly by ddH_2_O before added with pre-heated 1% (w/v) 2,3,5-triphenyltetrazolium chloride (TTC) staining solution. The incubation was lasted for 15 min at 37°C and protected from light, with which visual inspection was performed. The relative enzyme activities were measured spectrophotometrically (540 nm) by a DNS method as referred previously (Ma et al., 2016). As for the kinetic properties for native enzyme and variants, the *K*_m_ and *V*_max_ were determined by DNS method using inulin (2.9–52.1 mM) and sucrose (0.2–2.6 mM) as the substrates. All experiments were performed in triplicate and repeated three times. All curve fitting was performed using GraphPad Prism version 6 for Windows, GraphPad Software, La Jolla, CA, United States, www.graphpad.com.

### Fluorescence Spectra Analysis

Fluorescence spectra analysis was performed to evaluate the conformational change of variants as compared to WT. The buffer system of purified KcINU1, F90W, and M94A were change to 20 mM sodium acetate pH 4.5 through concentration and dilution using 10 kDa cutoff amicon ultrafiltration tubes (EMD Millipore, China) and the final protein concentrations were adjusted to 0.04 mg/ml. The fluorescence spectrums were recorded at a wavelength ranging from 290 to 400 nm on a FP-6500 fluorescence spectrophotometer (Jasco international Co., Ltd.) with the excitation wavelength of 280 nm and the spectrum of the sodium acetate buffer was recorded as the baseline.

## Results

### *In silico* Analysis

**FIGURE 1:**
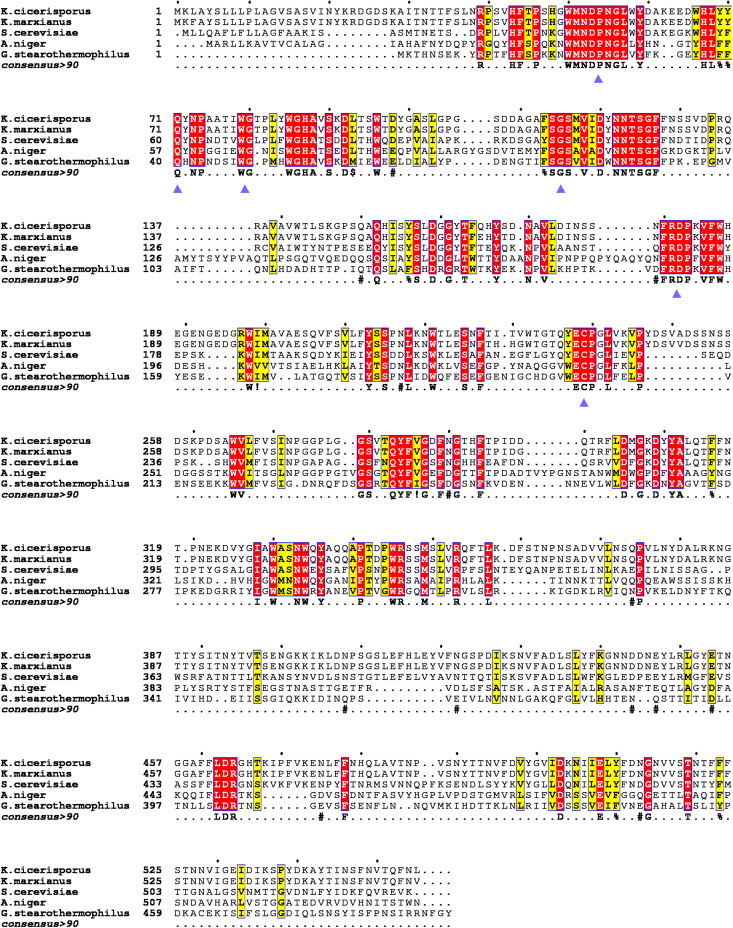
Multiple sequence alignments of the KcINU1 with other exo-inulinases of the GH32 family. The sequences of other protein used were *Kluyveromyces cicerisporus* exo-inulinase (GenBank accession number AAN32611), *Kluyveromyces marxianus* exo-inulinase (GenBank accession number AMX81520), *Saccharomyces cerevisiae* putative exo-inulinase (GenBank accession number CAA30457), *Aspergillus niger* exo-inulinase (GenBank accession number BAD01476.1), and *Geobacillus stearothermophilus* exo-inulinase (GenBank accession number BAC45010). The six conserved regions are marked with blue triangles below the consensus row. ! is anyone of residue I or V, $ is anyone of residue L or M, % is anyone of residue F or Y, # is anyone of residue NDQEBZ.

**FIGURE 2:**
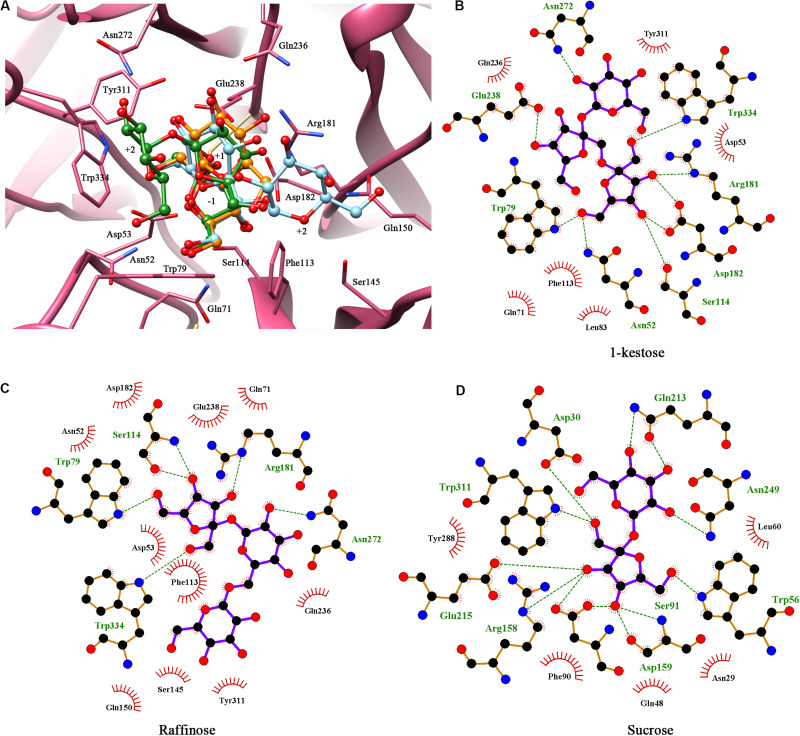
Molecular docking showed the potential binding interaction between KcINU1 and ligands. **(A)** Superimpose of the three ligands (1-kestose in green, raffinose in blue, and sucrose in orange) docked in the binding pocket of KcINU1 (colored in pink). **(B–D)** show the hydrogen bond interactions and hydrophobic interactions between KcINU1 and 1-kestose, raffinose, and sucrose, respectively. Interacting residues are labeled and shown as sticks. Hydrogen bonds are presented in green dashed lines.

Multiple sequence alignments of KcINU1 with several exo-inulinases from yeast, fungus, and bacterium were performed (Figure 1). The six conserved regions involved in substrate binding are marked with blue triangles which include WMNDPNG, Q, WG, FSGS, RDP, and ECP (Figure 1). To further investigate the substrate-enzyme interaction, KcINU1 was docked with 1-kestose, raffinose and sucrose (Figure 2A) using the Tripos’ Sybyl X-2.1.1 software package. The results show that the three ligands share comparable total scores, of which 1-kestose has the highest score, followed by raffinose, and sucrose is the last. The binding affinities of the three ligands to KcINU1 were further calculated using x-score (Wang et al., 2002) based on their best binding position obtained from docking calculation. The calculated *K*_*d*_ of 1-kestose is 1.58 μM which is slightly higher than raffinose (1.07 μM). Sucrose has the highest *K*_*d*_ (2.29 μM). Further analysis of the docking results showed that all three ligands bound to the similar substrate binding sites of the enzyme with the reducing end fructose residue located in the −1 position (Figures 2B–D). There is a strong hydrogen bond network stabilizing the sugar units at −1 and +1 position. The −1 fructose residue establishes hydrogen bonds with amino acid N52, S114, R181, D182, W79, and W334. At the +1 binding site, the fructose of 1-kestose establishes hydrogen bonds with E238, the glucose residue in raffinose forms hydrogen bond with N272, and the glucose unit in sucrose forms hydrogen bonds with Q213 and N249. The glucose residue in 1-kestose establishes a hydrogen bond with N272 and stabilized by hydrophobic interaction with W334. In contrast, the galactose residue in raffinose binds in an opposite direction, which is stabilized through hydrophobic interactions with F113 and S145.

Based on the *in silico* analysis, the roles of residue W79, F113, W334, and R181 in the enzyme-substrate interactions were evaluated by site-directed mutagenesis. Besides, a variant M117A in the FSGSM region was also constructed in order to determine the role of this locus.

### Expression and Purification of the Mutants

**FIGURE 3:**
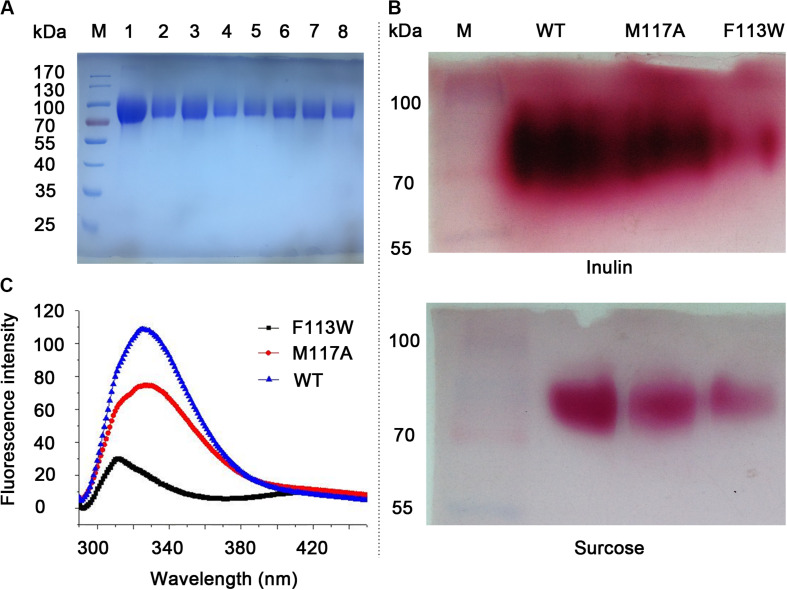
SDS-PAGE analysis, activity assay and fluorescence spectra of the WT and variants. **(A)** SDS-PAGE analysis of purified proteins. Lane M, pre-stained protein marker. Lane 1–8 is W79L, F113W, F113L, M117A, R181L, R181K, C239A, and W334L, respectively. **(B)** Zymogram analysis and TTC staining results for the WT, F113W, and M117A. Variants with no enzymatic activity toward inulin and sucrose were omitted from the figure. **(C)** Fluorescence spectra of the WT, F113W, and M117A.

All the variants (W79L, F113W, F113L, M117A, R181L, R181K, C239A, and W334L) were secreted expressed in *P. pastoris* X-33 cells after induction by methanol. After affinity chromatography Ni affinity column, the obtained variants were analyzed using SDS-PAGE, as shown in Figure 3A. The targeted proteins have clear bands around 90 kDa due to glycosylations (their predicted molecular mass were around 60.6 kDa) (Ma et al., 2016).

### Activity Assay and Kinetic Parameters Determination

The activities of KcINU1 and all variants were initially evaluated by zymogram assay using inulin and sucrose as the substrate. The results from zymogram assay showed reduced activity of F113W and similar activity of M117A as compared to wild-type (WT) enzyme (Figure 3B). Sample from other variants showed almost no stains on the PAGE thus not included in the figure. To further determine the relative activity of all variants, DNS method was conducted using 2% (w/v) inulin and 2% (w/v) sucrose as the substrate. In accordance with the zymogram assay, no activity were detected for all variants except the F113W which exhibited significantly reduced activity toward inulin (33.8 ± 3.3%) while retained the activity toward sucrose (96.2 ± 5.5%), and M117A which has similar activity on inulin (103.8 ± 1.3%) but an increased activity on sucrose (166.5 ± 12.0%) (Table 1). Further enzyme kinetic assay showed that the *K*_m_ for M117A (3.1 ± 0.3 mM) is similar to the WT (3.5 ± 0.3 mM), which is less than half of the value for F113W (7.7 ± 4.0 mM) (Table 1 and Supplementary Figure 1). This result is in accordance with the result of relative activity assay.

**TABLE 1:** Enzymatic activities and kinetic parameters of the KcINU1 and its variants.

Region	Mutants	Relative activity (%)		Kinetic parameters			
	
		Inulin	Sucrose	Inulin		Sucrose	
				
				*K*_m_ (mM)	*V*_max_ (U/mg)	*K*_m_ (mM)	*V*_max_ (U/mg)
	WT	100.0 ± 4.2	100.0 ± 3.0	3.5 ± 0.3	295 ± 17.9	30.0 ± 2.4	3445 ± 132.7
WG	W79L	–	–	–	–	–	–
FSGSM	F113W	33.8 ± 3.3	96.2 ± 5.5	7.7 ± 4.0	182.9 ± 75.4	94.0 ± 19.2	5770 ± 841.9
	F113L	–	–	–	–	–	–
	M117A	103.8 ± 1.3	166.5 ± 12.0	3.1 ± 0.3	298.1 ± 18.4	20.0 ± 3.5	4972 ± d336.4
RDP	R181L	–	–	–	–	–	–
	R181K	–	–	–	–	–	–
ECP	C239A	–	–	–	–	–	–
WQ	W334L	–	–	–	–	–	–

*–, Activity undetectable.*

To investigate the conformational change of variants F113W and M117A as compared to WT, the fluorescence spectra analysis was performed. The results showed there is a significant blue shift of the emission peak of F113W (313 nm) as compared to WT (326 nm). In contrast, the emission spectra profile of M117A is similar to the WT although a significant reduction of emission intensity is observed (Figure 3C).

## Discussion

In previous study, we have investigated the conserved residues (D30 and E215 in previous paper, in this paper numbered D53 and E238, respectively, including the 23 amino acids of the signal peptide) which involved in catalytic activity through sequences alignments (Ma et al., 2016). However, it should be noted that there was an aromatic region observed around the enzyme-catalyzed site in the GH32 family, which involves the sequences WMNDPN, WG, and W/FSGSAT. In the present study, multiple sequence alignments of KcINU1 with the other exo-inulinases indicated the presence of six conserved regions involved in substrate binding, such as 50WMNDPNG55, Q71, 79WG80, 113FSGS116, 181RDP183, and 238ECP240 (Figure 1). It was found that this hydrophobic conserved region played a very important role in maintaining the optimal and stable conformation of the binding site for the substrate. In particular, the W/FS conformation in the W/FSGSAT sequence may affect by the surrounding amino acids, which consequentially influent the binding of the fructose to the active site of the enzyme. Similar effects have been observed in *Cichorium intybus* Fructan 1-exohydrolase IIa that the S101 affects the conformation of W82 (Le Roy et al., 2008).

Further docking study demonstrated a strong hydrogen bond network stabilized the fructose residue at the −1 binding sites in all three ligands, similar as observed in *Schwanniomyces occidentalis* invertase (PDB code 3KF3) (Álvaro-Benito et al., 2010). The D53 at the −1 fructose binding site may serve as a nucleophile according to previous studies (Nagem et al., 2004; Álvaro-Benito et al., 2010; Ma et al., 2016). A strong hydrogen bond was observed between E238 and the glucose unit at the +1 binding site in the KcINU1-raffinose docking model (Figure 2C) similar to the observed interaction between D190 and the bound raffinose in *T. maritima* invertase (Alberto et al., 2006). In the KcINU1-kestose docking model, the fructose residue at the +1 binding site also forms a hydrogen bond with E238 (Figure 2B). The +2 sugar binding site for raffinose and 1-kestose are different. The glucose of 1-kestose forms interactions with N272, W79, and W334. In contrast, the galactose of raffinose binds through hydrophobic interactions with S145 and F113 which is in an opposite direction of the glucose unit of 1-kestose. Moreover, it is also different from the results observed in the invertase-raffinose complex structure that the galactose residue is suspended in the polar solvent and stabilized by the hydrogen network mediated with water molecules (Alberto et al., 2006). This reflects the possible existence of different +2 binding sites on KcINU1, and the site consisting of N272, W79, and W334 may be the main site when the enzyme interacts with inulin. It should also be noted that molecular docking only calculated the interaction between the protein and the ligand, no water molecule was taken into consideration.

Activity assay showed that all variants were inactive except the F113W and M117A which exhibited modified activity toward inulin and sucrose (Table 1). The diminished catalytic activity of W79L, R181L, R181K, C239A, and W334L demonstrated their important roles in substrate binding, which is in accordance with the *in silico* analysis. The W79 and F113 are located in the WG and FSGSM regions, which are equivalent to the W47 and W82 in AtcwINV1 (PDB code 2AC1), respectively. The catalytic activity of W79L was undetectable, which is similar to its counterpart W47 in AtcwINV1 that the W47L showed a significant increasing of *K*_m_ (600 times) as compared to WT (Le Roy et al., 2007). The W334 is located in the WQ region which is essential for stabilizing the bound substrate during enzyme-substrate interaction and its role was demonstrated with catalytic assay in this study. The R181 belongs to the RDP sequence and is equivalent to the R137 of the invertase from *T. maritime* (PDB code 1W2T) which forms an important hydrogen bond with the O4 of the glucose molecule and stabilizes the substrate (Alberto et al., 2004, 2006). However, in the KcINU1-kestose docking model, R181 forms a hydrogen bond with the −1 fructose instead of the +1 fructose. Mutation of R181 to Leu in KcINU1 may disrupt the potential hydrogen bond. Moreover, the side chain of R181 may play roles in neutralizing the negative electrostatic filed formed by D182 and E238, and the hydrophobic side chain of Leu may disrupt the electrostatic microenvironment, leading to the loss of activity. But to our surprise, the variant R181K also showed no activity although the introduced Lys shares similar properties with Arg such as the hydrogen bond formation ability. As observed in the complex structure of *Tm* invertase and raffinose (PDB code 1W2T), the R137 adopts an almost complete stretched conformation when forming a hydrogen bond with the ligand (Alberto et al., 2006). Therefore, the substitution of this Arg with a shorter side chain Lys may lead to the disruption of the hydrogen bond with the glucose unit of the ligand, instead unexpected hydrogen bond interactions or salt bridge interactions may form between the Lys and surrounding residues, and this could be the major cause of the inactivation of R181K of KcINU1. The C239 is located in the EC region, adjacent to the acid/base catalyst E238. Previous mutagenesis study in yeast SUC2 demonstrated the important role of C205 (the counterpart of C239) in the catalytic efficiency of the enzyme (Reddy and Maley, 1996). In the present study, it was observed that the activity of C239A could hardly be detected, which indicates its important role in maintaining the catalytic activity of the enzyme, and it may also contribute to stabilize the conformation of E238.

Interestingly, the variant F113W has a residual activity of 33.8 ± 3.3% and 96.2 ± 5.5% toward inulin and sucrose, respectively, and the *K*_m_ toward both substrates are significantly increased compared to WT (Table 1). In contrast, the F113L showed no activity to both substrates (Table 1). Proteins which contain aromatic amino acid residue, such as tryptophan and tyrosine, are known to have different fluorescence spectra when excited at 280 nm due to their side chain chromophores (Vivian and Callis, 2001). The maximum fluorescence emission wavelengths of the tryptophan and tyrosine residue in the protein are related to the polarity of the microenvironments. Therefore, conformational changes of a protein can be judged from the modulation of its emission profile. The results from present study showed a significant blue shift of the emission peak of F113W (313 nm) as compared to WT (326 nm) (Figure 3C). This may due to the fact that the aromatic ring of side chain of Phenylalanine provide important hydrophobic barrier to holding the fructose residue in place. The substitution of Phe with Trp may lead to a significant conformational change of the binding pocket. The bulky side chain of Trp may block the entrance of the binding pocket, and big substrates (such as inulin) are most affected. In contrast to the rigid conformation of the aromatic ring in Phe and Tyr, the side chain of Leu is highly flexible. It prone to protrude into the −1 binding sites and blocks the entry of fructose.

Unlike other residues investigated in this study, M117 is located in a β-sheet distant to the substrate binding pocket (Supplementary Figure 2) and much less conserved across species (Figure 1). Therefore, its role in inulinase activity needs to be clarified. Interestingly, substitution of this residue with Ala resulted in an almost unchanged activity toward inulin and an increased activity against sucrose (166.5 ± 12.0%) (Table 1). Enzyme kinetic assay also showed similar results that the *K*_m_ of M117A is comparable to WT on inulin while lower than WT on sucrose (Table 1). Further evaluation with fluorescence spectra showed a similar emission spectra profile of M117A as compared to the WT, although a significant reduction of emission intensity was observed which indicating the increased quenching effect (Figure 3C). This may reflect the conformational change of the variant caused by replacing M117 with Ala, which is conductive to the binding of smaller substrates (such as sucrose).

In summary, this work reports the *in silico* analysis and mutagenesis studies of KcINU1. The results from this study revealed that residues W79, F113, W334, R181, and C239 had played important roles in the enzyme-substrate binding processes. The W79, F113, and W334 had made up the aromatic hydrophobic region of substrate binding pocket of the enzyme. Therefore, by virtue of the Van Der Waals force and hydrophobic interaction between the aromatic ring and the sugar molecules, the substrate binds with the most stable and dominant conformations. Besides, the R181 was found to have interacted directly with the fructose residue via hydrogen bonds and had played a very important role in the substrate binding and recognition processes. The C239 had maintained the enzymatic activities of the enzyme by stabilizing the conformation of E238. This study elucidated the important role of aromatic amino acids in substrate binding in inulinase and facilitated future rational design of the enzyme for industrial applications.

## Data Availability Statement

The datasets presented in this study can be found in online repositories. The names of the repository/repositories and accession number(s) can be found in the article/Supplementary Material.

## Author Contributions

JM carried out the experiments and drafted the manuscript. TL contributed to the data analysis and manuscript editing and refinement. HT gave some good advice during the experiment. WL helped with protein expression. HY supervised this study. All authors read and approved the final manuscript.

## Conflict of Interest

The authors declare that the research was conducted in the absence of any commercial or financial relationships that could be construed as a potential conflict of interest. The reviewer PS declared past co-authorship with one of the authors TL to the handling editor.

## Footnotes

1 http://www.cazy.org/

2 http://www.cazy.org/GH32_structure.html

3 https://swissmodel.expasy.org/

4 http://www.rf-cloning.org/
