# Supplementary material for: The Important Roles Played in Substrate Binding of Aromatic Amino Acids in Exo-Inulinase From Kluyveromyces cicerisporus CBS 4857
Source: Front Mol Biosci. 2020 Sep 25;7:569797. doi: 10.3389/fmolb.2020.569797 (PMC7545266; doi:10.3389/fmolb.2020.569797)
Supplement: Supplementary file 1 [file Table_1.DOCX]

Supplementary Material

**Supplementary table 1.** Primers used for site-directed mutagenesis by RF-cloning.

| Primers | Sequences (5’→3’) |
| --- | --- |
| W79L-F | 5’-CACGATCTGGGGTACTCCATTGTAC**TTG**GGTCACGCTGTTTCC -3’ |
| W79L-R | 5’-GTCGTATGGAACCTTAACTAGACCTGGGCATTCGTATTGGGTACCAG-3’ |
| F113W-F | 5’-AGGTTCCGACGACGCTGGTGCG**TGG**AGTGGTAGTATGGTTATCG-3 |
| F113W-R | 5’-GTCGTATGGAACCTTAACTAGACCTGGGCATTCGTATTGGGTACCAG-3’ |
| F113L-F | 5’-AGGTTCCGACGACGCTGGTGCG**TTG**AGTGGTAGTATGGTTATCG-3’ |
| F113L-R | 5’-GTCGTATGGAACCTTAACTAGACCTGGGCATTCGTATTGGGTACCAG-3’ |
| M117A-F | 5’-CGCTGGTGCGTTCAGTGGTAGT**GCT**GTTATCGATTATAACAATACTT-3’ |
| M117A-R | 5’-GTCGTATGGAACCTTAACTAGACCTGGGCATTCGTATTGGGTACCAGT-3’ |
| R181L-F | 5’-TGTTGGACATCAACAGCTCCAACTT**CTT*G***GACCCTAAGGTGTTCTG -3’ |
| R181L-R | 5’-TTGGGGCTTGTTGGGCGTATTGCCAGTTAGAAGCCCATGCG-3’ |
| R181K-F | 5’-TGTTGGACATCAACAGCTCCAACTT**CAA*G***GACCCTAAGGTGTTCTGG-3’ |
| R181K-R | 5’-TTGGGGCTTGTTGGGCGTATTGCCAGTTAGAAGCCCATGCG-3’ |
| C239A-F | 5’-GCTGGACTGGTACCCAATACGAA**GCC**CCAGGTCTAGTTAAGG-3’ |
| C239A-R | 5’-TTGGGGCTTGTTGGGCGTATTGCCAGTTAGAAGCCCATGCG-3’ |
| W334L-F | 5’-GCTGGACTGGTACCCAATACGAATGCCCAGGTCTAGTTAAGG-3’ |
| W334L-R | 5’-TTGGGGCTTGTTGGGCGTATTG**CAA**GTTAGAAGCCCATGCG-3’ |

Forward (F) and reverse (R) sequences are shown with the mutations in bold underlined letters


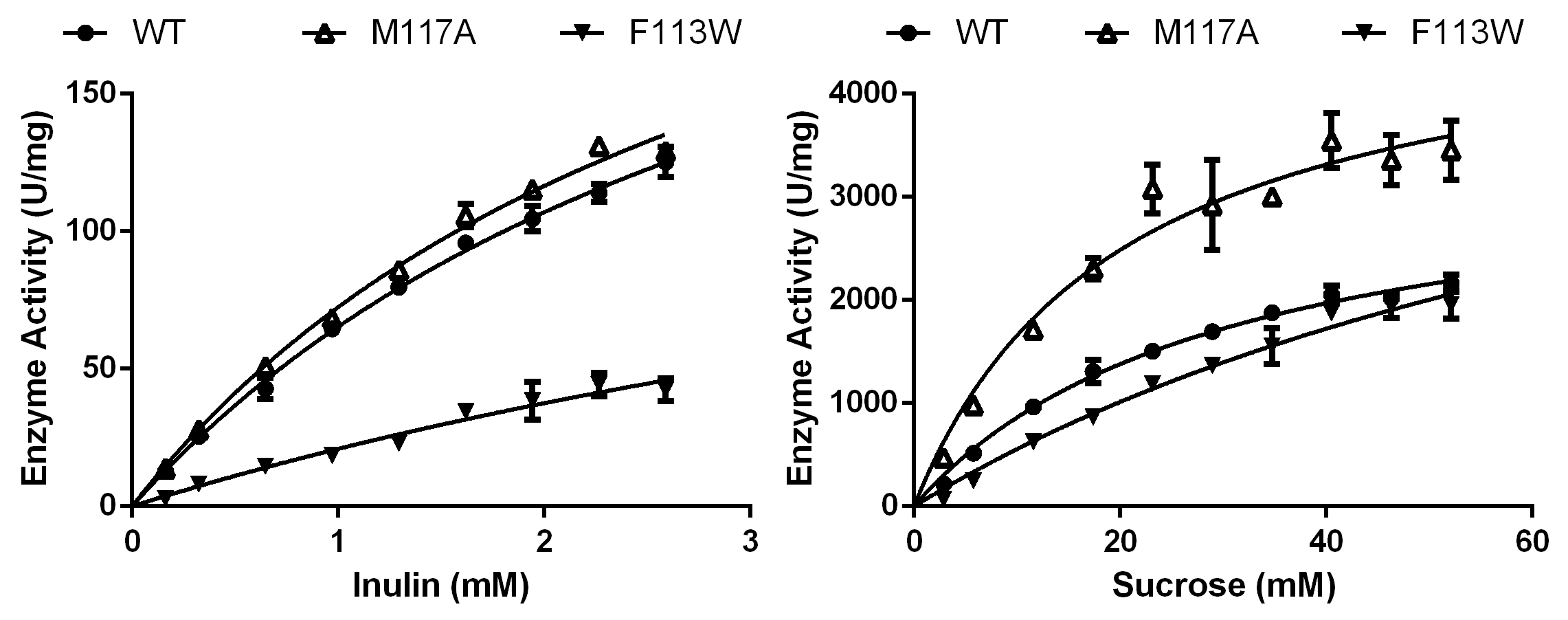


**Supplementary Figure 1.** The michaelis-Menten curve fitting by GraphPad Prism.

**
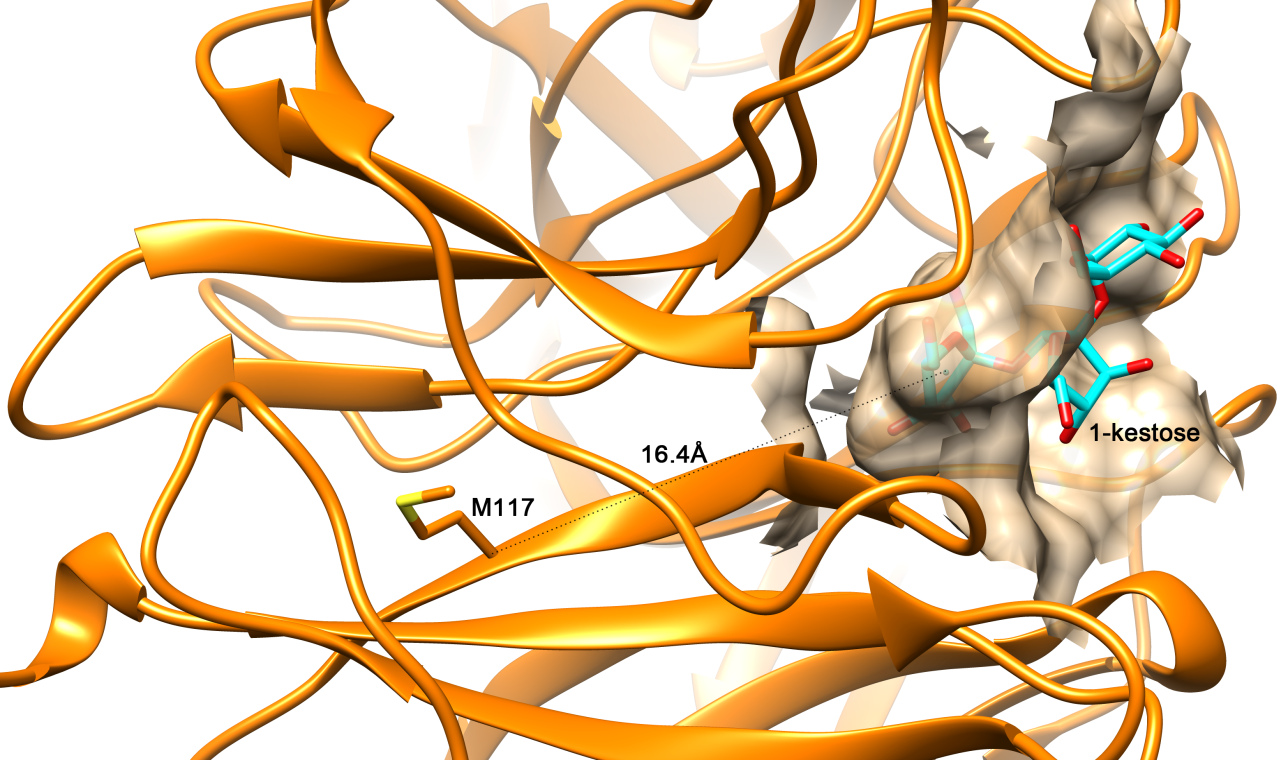
**

**Supplementary Figure 2.** The relative position of M117 and the docked 1-kestose in rKcINU1. The model of rKcINU1 is colored in orange, the bound 1-kestose is colored in cyan, and the surface of the binding pocket is colored in gray. The distance from the Cα atom to the centroid of the fructose in 1-kestose is labeled.
